# CD133: a stem cell biomarker and beyond

**DOI:** 10.1186/2162-3619-2-17

**Published:** 2013-07-01

**Authors:** Zhong Li

**Affiliations:** 1 Central Laboratory, the 10th People’s Hospital, Tongji University, 301 Middle Yanchang Road, Shanghai 200072, China; 2 Eastern Hepatobiliary Surgery Hospital/Institute, the Second Military Medical University, 225 Changhai Road, Shanghai 200438, China

## Abstract

Cancer stem cells (CSCs) or tumor initiating cells (TICs) contribute to tumorigenesis, metastasis, recurrence and chemoresistance. CD133, a pentaspan membrane glycoprotein, has been used as a stem cell biomarker for isolation of stem-like cells from a variety of normal and pathological tissues as well as cell lines since its discovery in 1999. Recent studies are focusing on the functionality of CD133. In this review, we summarize new insights into CD133 regulation and the involvement of CD133 in cell self-renewal, tumorigenesis, metastasis, resistance, metabolism, differentiation, autophagy, apoptosis, and regeneration.

## Introduction

Since CD133 was identified as a pentaspan transmembrane protein for human hematopoietic stem cells and mouse neuroepithelial cells [1-3], many studies have subsequently revealed that CD133 expression is associated with progenitor/stem cells, tumor, regeneration, differentiation, and metabolism. CD133 is one of key biomarkers for isolation and characterization of stem cells. Increasing evidence has shown that CD133 is not only a biomarker, but functions also in cell growth, development and tumor biology. Therefore, in this review, we will summarize the new functions of CD133.

CD133, also called Prominin-1, is a product of a single-copy gene on chromosome 4 (4p15.33) in human or chromosome 5 (5b3) in mice. Human CD133 is a transmembrane glycoprotein of 865 amino acids with a total molecular weight of 120 kDa. This protein consists of an N-terminal extracellular domain, five transmembrane domains with two large extracellular loops, and a 59 amino acids cytoplasmic tail [4]. It is selectively localized in microvilli and other plasma membrane protrusions [5,6]. In general, CD133 positive and CD133 negative cells display different characters. For example, 1) CD133^+^ and CD133^-^ glioma cells belong to independent cancer stem cell populations; 2) CD133^+^ glioma cells are derived from primordial CD133^-^ CSCs; 3) CD133^-^ CSCs retain their stem-like features as well as tumor initiation capacity, and can re-acquire CD133 expression in vivo; and 4) Both CD133^+^ and CD133^-^ CSCs have different expression profiles in transcriptional activities and extracellular matrix molecules [7,8].

### Regulation of CD133 expression

The CD133 expression is regulated by many extracellular or intracellular factors and represents changes of cell type with particular functions [9]. Griguer, et al. revealed that hypoxia, mitochondrial dysfunction or depletion of mitochondrial DNA induced a reversible up-regulation of CD133 expression [10]. Hypoxia-induced CD133 expression is also found in human lung cancer, pancreatic cancer and glioma cells [11,12]. Hypoxic condition increases hypoxia inducible factor 1α (HIF-1α) expression which inhibits the mammalian target of rapamycin (mTOR) C1 activity [12,13]. Increased HIF-1α induces the expansion of the CD133^+^ cells [11,12,14]. Pharmacological inhibition of mTOR with rapamycin greatly increases both the CD133^+^ populations and the expression of stem cell-like genes [14,15]. Enhancing mTOR activity by over-expressing Rheb significantly decreases CD133 expression, whereas knockdown of the mTOR yields an opposite effect [15].

Transforming growth factor β1 (TGFβ1) is identified to be capable of up-regulating CD133 expression specifically within the Huh-7 hepatocellular carcinoma (HCC) cell line in a time- and dose-dependent manner [16]. TGFβ1 inhibits DNA methyltransferases (DNMT) 1 and DNMT3β expression and subsequently induces the demethylation of promoter-1 of CD133 [16]. Analysis of Toll-like receptors (TLR) in colorectal cancer (CRC) reveals that TLR7 and 8 increase in CD133^+^ cells in CRCs [17]. Both TLRs and chemokines activate NF-κB signaling in cancer stem cells [18,19]. Therefore, CD133 expression may play an important role in communication through membrane receptors.

MicroRNA (miRNA) profiling has revealed that several miRNA are involved in regulation of CD133 expression in a variety of cells. By analyzing miRNA expression profiling of CD133^+^ and CD133^-^ cells from human HCC clinical specimens and cell lines, Ma, et al. has identified elevated miR-130b in CD133^+^ HCC TICs [20]. Forcing expression of miR-130b in CD133^-^ cells enhances their chemoresistance, self-renewal and tumorigenicity in vivo. But upregulation of miR-125b inhibits the invasion of CD133^+^ primary glioblastoma cells [21]. In addition, miR-142-3p [22], miR-199b-5p [23], miR-143, miR-145 [24], and miR-150 [25] show inhibition of the colony-forming ability and tumor sphere formation of CD133^+^ cells. However, most of these miRNAs exhibit indirect regulation of CD133 expression. A specific miRNA targeting CD133 expression has not been identified yet.

CD133 expression is also regulated by epigenetic factors. Methylation of the CD133 promoter represses CD133 gene transcription. Demethylation of the CD133 gene has been found in a variety of human tumors including colorectal cancer [26], gastric carcinoma [27], gliomas and glioblastoma [28,29], HCC [30], and ovarian cancer [31] and so on. TGFβ1 induced CD133 expression via demethylation of CD133 promoter-1 in Huh-7 cells [16]. Upregulation of CD133 is in CRC that exhibits a hyperactivated Ras-Raf-MEK-ERK pathway secondary to mutations in K-Ras or B-Raf [32].

### CD133 in cell self-renewal and tumorigenesis

Freshly isolated CD133^+^ cancer cells from colorectal cancer, gallbladder carcinoma, HCC, ovarian cancer and other tumors gave rise to long-term tumor spheroids and xenograft tumors in immunodeficient mice [20,33-35]. The underlying mechanisms involved in regulation of self-renewal in HCC may depend on the Akt/PKB and Bcl-2 pathway [36]. Using the genome-wide microarray analysis, Tang et al. revealed that a significant interleukin-8 (IL-8) signaling network was activated in CD133^+^ liver TICs obtained from HCC clinical samples and cell lines responsible for self-renew, tumor angiogenesis, and tumorigenesis [37]. C-terminal cytosolic domain of CD133 is phosphorylated by Src-family kinases as determined by mass spectrometry and site-directed mutagenesis. Tyrosine-828 and the nonconsensus Tyrosine-852 are the major tyrosine phosphorylation sites [38]. T-828 phosphorylation of CD133 mediates activation of PI3K/Akt pathway in glioma stem cells through interaction with p85 regulatory subunit [39]. Silencing of CD133 impairs the self-renewal and tumorigenic capacity of tumor cells [40].

Although both CD133^+^ and CD133^-^ cells are capable of tumor initiation in the nonobese diabetic/severe combined immunodeficient (NOD/SCID) mice, most of CD133^+^ tumor subpopulations form colonospheres in an in vitro culture and retain long-term tumorigenic capacity in a NOD/SCID serial xenotransplantation model [41]. Upstream molecules in Akt and mitogen-activated protein kinase (MAPK) pathways are preferentially activated in CD133^+^ colon cancer cells [42]. Ras and its downsteam effectors such as ERK, JNK, PI3K, p38K, and RalA are also significantly activated in CD133^+^ human primary malignant peripheral nerve sheath tumor [43]. Stemness genes, octamer biding transcription factor 3/4 (OCT4) and/or SRY-box containing gene 2 (SOX2), have been found to bind to the P1 promoter region of CD133 gene loci and ectopic OCT4 or SOX2 expression triggers the CD133P1 activity in the lung cancer cell lines N417, H358, and A549 [44]. Therefore, CD133 expression is essential for self-renewal function and tumorigenesis in certain cell types.

### CD133 and metastasis

Increasing evidence indicates that a subset of tumor cells contributing to metastasis has the properties of CSCs or TICs. CD133^+^ cells are higher in liver metastasis than in primary colorectal tumors [45]. Compared with CD133^+^CXCR4^-^ cells, CD133^+^CXCR4^+^ cancer cells have a high metastatic capacity in vitro and in vivo and undergoes epithelial-mesenchymal transitions (EMT)[45]. CD133^+^CD44^+^ cancer cells have been characterized in several highly metastatic tumors, such as CRCs [46-48], HCCs [49,50], pancreatic cancer [51], gallbladder carcinoma [52], lung adenocarcinomas [53] and gastric cancer [54]. Immunohistochemical study of human HCC specimens reveals that the number of CD133^+^ CD44^+^ HCC cells is increased and associated with portal vein invasion [49]. In colorectal cancer with early liver metastases, co-expression of CD133 and CD44 is significantly higher when compared to those without early liver metastases [48]. Knockdown of CD133 in hepatocarcinoma PLC/PRF/5 and HCT116 cells results in decreased expressions of matrix metalloproteinase (MMP)-2, a disintegrin and metalloproteinase (ADAM)9 [55,56]. These lead to decreased invasion as demonstrated in an in vitro system [55,56]. In addition, chemokine CCL5 and its receptors, CCR1, CCR3 and CCR5, are found to be upregulated in CD133^+^ cancer stem-like cells from ovarian cancer [57]. Blocking of CCL5, CCR1 or CCR3 effectively inhibits the invasive capacity of these cells via inhibition of NF-kappaB and MMP9 secretion [57]. Therefore, CD133^+^ TICs may confer metastatic potential to their progenies.

### CD133 and chemo- and radio-resistance

CD133 positive cells show a high degree of chemoresistance. CD133^+^ lung cancer cells exhibit drug resistance [58]. Isolated CD133^+^ CSCs from human oral squamous cell carcinoma are substantially resistant to standard chemotherapy [59]. Ectopic overexpression of CD133 in rat C6 glioma cells leads to significant reluctance to undergo apoptosis from camptothecin and doxorubicin treatments [60]. Chemoresistant CD133^+^ cells usually have the upregulation of ATP-binding cassette (ABC) transporter [52,60]. Since ATP-binding cassette sub-family B member 5 (ABCB5)- mediated doxorubicin efflux [61], suppression of ABCB5 sensitizes the cells to doxorubicin uptake and apoptosis [62]. Moreover, CD133-expressing liver cancer cells following radiation exposure show higher activation of MAPK/PI3K signaling pathway and reduction in reactive oxygen species levels compared to CD133^-^ cells. The irradiated CD133^+^ cell induces an increase of tumor formation in an in vivo xenograft model compared to the CD133^-^ group, suggesting that CD133 contributes to radioresistance in HCC [63]. Treatment of unsorted HCC cells with anticancer drugs in vitro also significantly enriches the CD133^+^ subpopulation [36].

### CD133 and metabolism

In epithelial cells, CD133 is found in microvilli, the primary cilium and the midbody [64]. This membrane protein has been found to be released from apical midbodies and the primary cilium of neuroepithelial cells as a whole or in part, into the extracellular space, yielding the CD133-enriched membrane particles found in the neural tube fluid [65]. Intriguingly, the release of these particles has been implicated in (neuro)epithelial cell differentiation [64]. CD133 is selectively associated with microvilli and largely segregated from the membrane subdomains containing placental alkaline phosphatase [66]. CD133 is also a cholesterol-interacting membrane protein responsible for the generation of plasma membrane protrusions, their lipid composition and organization as well as the membrane-to-membrane interactions [67]. Unraveling that CD133 inhibits transferrin uptake and AC133 antibody downregulates this uptake [68] further indicates the involvement of CD133 in cell metabolism.

Hexokinase II is a key enzyme in the glucolytic pathway. Its gene expression and enzymatic activity are lower in CD133^+^ than in CD133^-^ hepatoma BEL-7402 [69]. Pancreatic cancer patients with low expression of hexokinase II have significantly shorter survival than those with higher expression [70]. Higher expression of hexokinase II is associated with advanced tumor grade and higher stage as well as higher mortality in HCC [71].

β-galactoside α2,6-sialyltransferase (ST6Gal-I) adds an α2-6-linked sialic acid to the N-glycans of CD133 membrane proteins that may stabilize CD133 [72]. ST6Gal-I has been reported to be upregulated in human colon cancer, induced pluripotent stem (iPS) cells and CSCs [73]. CD133 has eight N-glycosylation sites on its extracellular loops [4]. Lectin binding assay for cell surface glycan epitopes and microarray analysis for expression of N-glycan biosynthesis-related genes demonstrate that over 10% difference between CD133^+^ and CD133^-^ hematopoietic stem and progenitor cells (HSPC) [74]. Biantennary complex-type N-glycans are enriched in CD133^+^ cells that have the overexpressed mannosyl (α-1,6-)-glycoprotein β-1,2-N-acetylglucosaminyltransferase (MGAT) 2 and underexpressed MGAT 4 [74]. Moreover, the amount of high-mannose type N-glycans and terminal α2,3-sialylation is increased in CD133^+^ cells [74]. N-glycosylation of CD133 is thought to be associated with cell differentiation [75] and promoted by hypoxia [76]. In addition, silencing CD133 reduces the glucose uptake [77], indicating that CD133 expression may be responsible for energic metabolism and the survival of CSCs.

Further analysis of signaling pathways in CD133^+^ and CD133^-^ cells has found that freshly isolated CD133^+^ cells from benign prostate tissue show expression of transcripts associated with cell development, ion homeostasis and cell communication, whereas profiling of CD133^-^ cells revealed gene transcripts related to cell proliferation and metabolism [78]. In human cord blood-derived CD133^+^ cells, 690 transcripts are differentially expressed in CD133^+^ and CD133^-^ cells. Of these, 393 are increased and 297 are decreased in CD133^+^ cells in which that the highest overexpression genes are associated with metabolism, cell communication, and development [79]. Transcriptomic profiling of sorted CD133^+^ and CD133^-^ cells from human glioblastoma multiforme reveals a CD133 gene expression signature composed of 214 differentially expressed genes [80]. Moreover, comparison of transcripts in CD34^+^ and CD133^+^ cells reveals that CD133^+^ cells have higher numbers of up-regulated genes than CD34^+^ cells. The uniquely expressed genes in CD34^+^ or CD133^+^ cell populations are associated with different biological processes: CD34^+^ cells overexpress many transcripts associate with development, while CD133^+^ cells express genes associated with chromatin architecture, DNA metabolism, and cell cycle [81].

### CD133 and differentiation

CD133 is expressed on both CSC and differentiated tumor cells. CD133 is possibly folded as a result of differential glycosylation to mask specific epitopes [75]. Although both CD133^+^ and CD133^-^ cells derived from primary glioblastomas show similar tumorigenicity in nude mice, there are 117 genes differentially expressing in these two subtypes [82]. Observation of CD133 expression in several neuroblastoma cell lines/tumor samples has shown that CD133 represses neurite extension and the expression of differentiation marker proteins, but accelerates cell proliferation, anchorage-independent colony formation and in vivo tumor formation of neuroblastoma cells [83]. Platelet-derived growth factors in the presence of a cytokine cocktail suppress ex vivo expansion of umbilical cord blood CD133^+^ cells and enhance their differentiation into megakaryocytic progenitor cells in a dose- and time-dependent manner [84]. Consistent with rapamycin increasing CD133 expression, mTOR inhibition severely blocks the differentiation of CD133^+^ to CD133^-^ liver tumor cells [15]. Interestingly, single-cell culture experiments have revealed that CD133^-^ liver tumor cells are capable of converting to CD133^+^ cells and the inhibition of mTOR signaling substantially promotes this conversion [15]. However, we should also note that CD133 expression and post-translational modification are dynamic and reversible that are dependent on cell microenvironment and physiological regulation [85].

### CD133 and autophagy

Autophagy as a key homeostatic process of cytoplasmic degradation and recycling is associated with the status of tumor cells. The shift of CD133 subcellular localization from the cytoplasm to the plasma membrane leads to the alternation of its functions [40]. CD133 has been shown to affect the clathrin-endocytosis process [68]. We have found that CD133 expression promotes glucose uptake and autophagosome formation in the glucose deprivation [77]. Immunofluorescence and time-lapsed confocal techniques demonstrate co-locolization of CD133 with an autophagy marker, microtubule-associated protein light chain3 (LC3) and a lysosome marker. CD133-mediated functions are beneficial for CSC survival. Knockdown of CD133 by siRNA attenuates production of LC3-II while the expression of autophagy associated genes (Atg9, Atg5/Atg12, and beclin-1) is not affected [77]. We speculate that CD133-mediated autophagy may be involved in the membrane-mediated phagophore formation.

### CD133 and apoptosis

CD133^+^ cell population demonstrates significant resistance to TGF β- and TNF-related apoptosis-inducing ligand (TRAIL)- induced apoptosis compared with CD133^-^ cells [86,87]. High expression of FLICE-like inhibitory protein (FLIP), an inhibitor of the extrinsic apoptotic pathway, in CD133^+^ cells is thought to be associated with the resistance to the apoptosis induced by TRAIL [88]. In addition, CD133^+^ population has grater resistance to staurosporine-induced apoptosis than CD133^-^ population [89] and stress-induced apoptosis [90]. Targeting CD133 by its antibody leads to cell death via attenuation of autophagy and promotion of apoptosis in HCC cells [77].

### CD133 and regeneration

CD133^+^ cells that are isolated from bone marrow, cord blood, and peripheral blood have been tested in both animal models and clinical trials in an attempt to repair the injured tissues with the pluripotent of CD133^+^ cells [91]. The cell lines derived from human endothelial progenitor cells and cord blood undergo in vitro pre-angiogenic process, form pseudovessel structures and present an accelerate angiogenesis in hypoxic conditions [92]. Cells isolated from the peripheral blood using CD133 antibodies have been shown through a mouse spinal cord injury model as being able to enhance angiogenesis, astrogliosis, axon growth and functional recovery. In contrast, the administration of CD133^-^ cells fails to promote axon growth and functional recovery, but moderately enhances angiogenesis and astrogliosis [93]. When CD133^+^ cells embedded in atelocollagen gel into a silicone tube is used to bridge a 15-mm defect in the sciatic nerve of athymic rats, sciatic nerves are structurally and functionally able to regenerate within 8 weeks and the transplanted CD133^+^ cells are differentiated into Schwann cells [94]. In a muscle injury rat model, granulocyte colony stimulating factor-mobilized peripheral blood CD133^+^ cells are differentiated into endothelial and myogenic lineages [95]. In addition, autologous bone marrow-derived CD133^+^ stem cell therapy has been used in clinical trials for patients with chronic total occlusion and ischemia [96], myocardial infarction [97], hepatic fibrosis [98], and liver regeneration [99]. The CD133^+^ cells have also been used for cardiac stem cell therapy [100] and bone regeneration [101]. Better application and expansion of CD133^+^ cells may yield tremendous benefits for tissue engineering.

### Perspective

**Figure 1:**
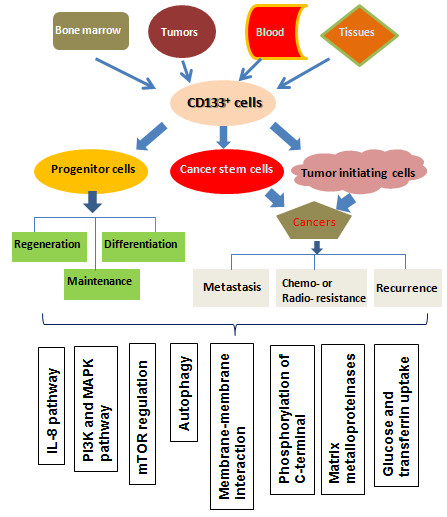
**Functional outline of CD133**^**+ **^**cells.** The number of CD133^+^ cells is maintained in a relative constant in bone marrow, blood, different tissues and even tumors. When cells or tissues are damaged by chemical, physical or mutational causes, CD133^+^ progenitor or stem cells are activated to self-renew, proliferate and differentiate in order to repair the damage. CD133^+^ CSCs or TICs are responsible for tumor metastasis, chemo- or radio-resistance and recurrence. CD133 expression is dynamic and reversible in response to the changes of cell microenvironment. CD133 is involved in diverse cellular processes, including glucose and transferrin uptake, autophagy, membrane-membrane interaction, and matrix metalloproteinase functions. IL-8 pathway, mTOR, PI3K and MAPK pathways are preferably activated in the CD133^+^ cells.

To better understand how to modulate the stem cells, particularly cancer stem cells, we have to identify specific biomarkers. Extensive studies of CD133 in different fields have provided new insights into the diverse CD133 functions (Figure 1). However, it remains a challenge to integrate the available expression, regulatory, structural, and functional data for this fascinating protein [102].

## Competing interests

The author declares that he has no competing interests.
